# Depot buprenorphine as an opioid agonist therapy in New South Wales correctional centres: a costing model

**DOI:** 10.1186/s12913-022-08687-8

**Published:** 2022-11-08

**Authors:** R. Ling, B. White, J. Roberts, M. Cretikos, M. V. Howard, P. S. Haber, N. Lintzeris, P. Reeves, A. J. Dunlop, A. Searles

**Affiliations:** 1 Hunter Medical Research Institute, University of Newcastle, Lot 1 Kookaburra Cct, New Lambton Heights, NSW 2305 Newcastle, Australia; 2 School of Medicine and Public Health, Faculty of Health and Medicine, University of Newcastle, Callaghan, NSW Australia; 3 Drug and Alcohol Clinical Research and Improvement Network, Sydney, NSW Australia; 4 Drug Health Services, Edith Collins Translational Research Centre, Sydney Local Health District, Camperdown, NSW Australia; 5 Speciality of Addiction Medicine, Faculty of Medicine and Health, The University of Sydney, Camperdown, NSW Australia; 6 Justice Health and Forensic Mental Health Network, Malabar, NSW Australia; 7 Centre for Population Health, NSW Ministry of Health, St Leonards, NSW Australia; 8 Corrective Services New South Wales, Sydney NSW, Australia; 9 Drug and Alcohol Services, South Eastern Sydney Local Health District, Surry Hills, NSW Australia; 10 Drug & Alcohol Clinical Services, Hunter New England Local Health District, Newcastle, NSW Australia

**Keywords:** Costing study, Opiate agonist therapy, Economics

## Abstract

**Background:**

In 2019 daily liquid methadone and sublingual buprenorphine-naloxone were primary opioid agonist treatments for correctional centres in New South Wales, Australia. However, both had significant potential for diversion to other patients, and their daily administration was resource intensive. An alternative treatment in the form of subcutaneous depot buprenorphine became a viable option following a safety trial in 2020 – the UNLOC-T study. Depot preparation demonstrated advantages over current treatments as more difficult to divert and requiring fewer administrations. This paper reports the results of economic modelling of staffing costs in medication administration comparing depot buprenorphine, methadone, and sublingual buprenorphine provision in UNLOC-T trial facilities.

**Methods:**

The costing study adopted a micro-costing approach involving the synthesis of cost data from the UNLOC-T clinical trial as well as data collected from Justice Health and Forensic Mental Health Network records. Labour and materials data were collected during site observations and interviews. Costs were calculated from two payer perspectives: a) the New South Wales (state) government which funds custodial and health services; and b) the Australian Commonwealth government, which pays for medications. The analysis compared the monthly-per-patient cost for each of the three medications in trial-site facilities during July 2019. This was followed by simulation of depot buprenorphine implementation across the study population. Costs associated with medical assessment and reviews were excluded.

**Results:**

The monthly-per-patient New South Wales government service costs of depot buprenorphine, methadone and sublingual buprenorphine were: $151, $379 and $1,529 respectively while Commonwealth government medication costs were $434, $80 and $525. The implementation simulation found that service costs of depot buprenorphine declined as patients transitioned from weekly to monthly administration. Costs of treatment using the other medications increased as patient numbers decreased alongside fixed costs. At 12 months, monthly-per-patient service costs for depot buprenorphine, methadone and sublingual buprenorphine—which would be completely phased out by month 13—were $92, $530 and $2,162 respectively.

**Conclusions:**

Depot buprenorphine was consistently the least costly of the treatment options. Future modelling could allow for dynamic patient populations and downstream impacts for participants and the state health system.

**Trial registration:**

ACTRN12618000942257. Registered 4 June 2018.

**Supplementary Information:**

The online version contains supplementary material available at 10.1186/s12913-022-08687-8.

## Background

In 2019, liquid methadone (> 90%) and sublingual buprenorphine-naloxone were the primary opioid agonist treatments used in correctional centres in New South Wales. The observed benefits of providing opioid agonist treatment (OAT) to people with opioid dependence in custodial settings include reduced drug use in prison and reduced mortality in prison [1] and on release [2]. Despite these benefits, there remains significant under-treatment of patients with opioid use disorder and suboptimal provision of OAT in custodial settings worldwide [3, 4]. Key reasons for under-treatment stem from capacity constraints in the system for medical and nursing staff [5], concern regarding diversion of opioid medications within prison populations [6], and the need for more intensive supervision than in community settings, particularly for sublingual buprenorphine [7–9].

A comparison of figures sourced from the 2018 National Opioid Pharmacotherapy Statistics Annual Data collection [10] and Australian Bureau of Statistics data on prison populations [11] suggests that in 2018, NSW correctional centres managed approximately 7–8% of all patients on OAT in NSW and approximately 12% of the adult population in prison was on OAT. Despite having one of the largest prison-based OAT programs in the region [4] and the recognised benefits of OAT, program upscaling was not previously possible due to resource constraints, with OAT delivery balanced against the competing demands of other health service needs.

In November 2018, CAM2038—a modified release depot buprenorphine formulation available for weekly or monthly administration -– was registered as Buvidal® by the Therapeutic Goods Administration in Australia [12] and recommended by the Pharmaceutical Benefits Advisory Committee in November 2019 [12]. Another formulation of depot buprenorphine available for monthly dosing only – Sublocade®—received regulatory approval in early 2020 and government subsidy in May 2020 [13, 14]. Sublocade® is available for in the U.S. and Canada; Buvidal® is available in several countries in Europe. Depot buprenorphine (depot BPN) is a subcutaneous injection of buprenorphine prepared with excipients allowing gradual release over one week or month depending on the formulation [12]. Community-based studies have found good retention in depot BPN treatment and high patient satisfaction [15, 16].The depot formulation required only relatively infrequent weekly or monthly individual administration inside a consultation room, in contrast to daily administration of methadone and sublingual buprenorphine-naloxone (SL BPN-NLX) to groups of patients outside correctional facility clinics via a ‘dosing window’. Diversion of depot BPN has not been previously studied and therefore suitability for use in correctional settings warranted further investigation.

A NSW Health sponsored study into the safety and feasibility of depot BPN in NSW correctional settings commenced in late 2018. The ‘Understanding NSW Long-acting Opioids in Custody-Treatment’ (UNLOC-T) study was a non-randomised, open-label two-arm study which compared patients initiated on depot BPN to patients already receiving oral methadone [17]. Briefly, this open label, non-randomised trial recruited 129 men and women, aged ≥ 18 years of various security classifications with a diagnosis of moderate to severe DSM-5 opioid use disorder currently serving a custodial sentence of ≥ 6 months within one of seven NSW correctional centres located across metropolitan and regional areas of NSW. Patients not in OAT at recruitment commenced depot buprenorphine (*n* = 67); patients already stable on oral methadone treatment were recruited to the comparison arm (*n* = 62). Depot buprenorphine (CAM2038 weekly for 4 weeks then monthly) was directly compared against daily oral methadone. Safety was assessed by adverse event (AE) monitoring and physical examinations at every visit. Participants were administered a survey assessing self-reported diversion and substance use at baseline and weeks 4 and 16. The trial results demonstrated that treatment retention and outcomes were comparable to results observed in community settings as well as for other opioid agonist treatment used in custodial settings, without increased risk of diversion. The UNLOC-t study found that depot BPN showed significant reduction in use of non-prescribed opioids and the use of any injecting drugs [17]. Further details of the UNLOC-T trial have been reported elsewhere [17].

The unique custodial and safety-related challenges of providing health care in correctional settings have significant resources implications. While most patients attend a health clinic in small groups to receive supervised daily treatment, some individuals required higher levels of security. For example, a small number of patients, in segregation, require escorts of two or more officers to attend the health clinic. Others are unable to attend the clinic for security reasons, requiring health staff to deliver treatment via special ‘satellite’ clinics located closer to cells. The highly resource intensive nature of OAT administration in these settings required consideration of factors distinct from community settings [18].

Initial evidence from community studies suggests higher acquisition costs for prolonged release forms of buprenorphine (injectable and implantable) than for methadone or SL BPN-NLX. However, there are potentially favourable cost advantages in administration, downstream health care and justice costs, and patient quality of life [19, 20].

The economic costs and cost-effectiveness of methadone in custodial settings has been reported previously [21, 22]. Warren et al. modelled the incremental cost effectiveness of the methadone program in the NSW public correctional system, comparing the cost and outcomes for patients from the perspective of the NSW government. They based their analysis on the results of a randomised controlled trial. The study found an incremental cost (compared to no methadone treatment) of $7,085 per inmate per year (2021-22AUD) [23, 24]. Costs included government administration, methadone administration, onsite labour costs (correctional and medical staff), methadone, disposables and pharmacy costs including transport [22].

Horn et al.’s [21] 2018 US six-week study estimated the cost of a methadone program in an urban facility from the service provider perspective. The authors found an average weekly cost per patient of $161 ($8,372 per year) (2021–22 AUD). Costs included on site medical, administration and pharmacy costs; facilities maintenance, program equipment including laboratory and computers. Although there is a formative literature on depot BPN, gaps remain in knowledge regarding the costs and benefits of depot BPN in prison settings.

Based on the UNLOC-T clinical trial of depot BPN [17], we undertook an economic costing study to measure and compare the costs of administering three alternate forms of OAT: a) depot BPN; b) methadone; and c) SL BPN-NLX, across seven correctional centres in NSW, Australia. Additionally, cost-modelling was employed, extrapolating the trial findings, to provide decision makers with information about the relative resource use requirements to implement depot BPN, compared to other OAT, across NSW prisons.

## Methods

### Research ethics and trial registration

This research trial was approved in 2018 by human research ethics committees of Justice Health and Forensic Mental Health Network (JHFMHN), the Aboriginal Health and Medical Research Council (AH&MRC), and Corrective Services New South Wales (CSNSW) (Protocol JHFMHN File No G561/17 & HREC/18/JHFMHN/3) including this economic evaluation. The trial was registered to the Australia New Zealand Clinical Trials Registry (ANZCTR) as trial, ACTRN12618000942257, 4/06/2018. The research was funded by the NSW Ministry of Health. The study adhered to the Consolidated Health Economic Evaluation Reporting Standards (CHEERS) (Additional Files 2).

### Costing study design

The costing study adopted a micro-costing approach and involved the synthesis of cost data from the UNLOC-T clinical trial as well as data collected from JHFMHN records [25] to reflect the treatment pathway for patients in pre-existing OAT programs at the trial centres per the approach employed by Warren et al. [22]. Micro-costing, also known as 'bottom up' costing is a strategy for calculating total costs by collating and summing the costs of individual program components, no matter how small. It is a most accurate approach given its comprehensive nature [26]. The study calculated and reports monthly-cost-per-patient, compared across each comparator group.

### Study comparisons, time horizon and perspective

The study included three comparator cohorts: a) UNLOC-T trial depot BPN participants; b) all pre-existing methadone patients at each UNLOC-T centre; and c) all pre-existing SL BPN-NLX patients at each UNLOC-T centre. Study patient populations were observed for the month of July 2019, the midpoint of the UNLOC-T trial. The study centres were all managed by CSNSW [27] and comprised one minimum-security, two maximum security and four mixed security facilities. The largest centre was a mixed-security facility with a population of over 600 at the end of the 2018 financial year [25]. Table 1 presents an overview of the trial centres by patient and treatment administration.

**Table 1:** Trial facilities by patients and treatment administrations

				**Study Patients**					**Administrations July 2019**		
Site	Gender	Security	Total Inmates 2019 Census (n)^a^	Depot (on Monthly Administrations) (n)^b^	Depot (on Weekly Administrations) (n)^b^	Depot Total (n)	Methadone (n)^c^	SBL BPN (n)^c^	Depot (n)^b^	Methadone (n)^c^	SBL BPN (n)^c^
Inner Metro 1	Male	Min	610	4	4	8	33	5	11	1,023	155
Inner Metro 2	Male	Min/Med/Max	451	5	3	8	16	1	12	496	31
Outer Metro 1	Female	Min/Med/Max	212	4	0	4	21	3	4	651	93
Regional 1	Male	Max	398	0	6	6	32	5	20	992	155
Regional 2	Male	Min/Max	526	10	0	10	32	12	10	992	372
Regional Remand	Male	Min/Med	627	0	5	5	53	13	18	1,643	403
Regional 3	Male	Max	420	9	0	9	52	1	9	1,612	31
**Totals**			**3244**	**32**	**18**	**50**	**239**	**40**	**84**	**7409**	**1240**

^a^NSW Inmate Census 2019 [25]

^b^Trial data. The number of depot administrations is greater than the number of patients because there was also weekly and titration dosing

^c^Median of monthly OAT reports: August 2018 to July 2019 [27]

The analyses were conducted from the perspectives of the NSW government, which was responsible for the delivery of OAT services and the Commonwealth government as the funder of OAT medication. The study did not include a patient perspective as there were no ‘out-of-pocket’ health care costs. All costs are reported in 2021–22 Australian dollars ($AUD) [23, 24].

### Opioid agonist treatments (OAT) included in the study

Depot buprenorphine (BPN) was supplied in pre-filled syringes. Monthly buprenorphine preparations included doses of 64, 96, 128 or 160 mg. Weekly buprenorphine preparations included doses of 8, 16, 24 and 32 mg. Treatment administration occurred during a nurse consultation where patients were questioned about their health and tolerability of the medication.

Sublingual buprenorphine (SL BPN-NLX) is supplied as a sublingual film, packaged in boxes of 28, containing films of one of two strengths: 2 mg and 8 mg. Individual doses are prepared by JHFMHN clinic nurses according to patients’ individual prescriptions administered daily.

The trial regimen specified that depot buprenorphine treatment be initiated with a 4 mg test-dose of SL BPN-NLX film (Day 0), followed with a single dose of depot BPN weekly 16 mg injection on Day 1 [16]. Patients received a total of four doses of depot BPN weekly (day 1, week 1, week 2 and week 3) before transferring to three doses of depot BPN monthly (week 4, week 8 and week 12). If required, patients could receive additional 8 mg depot BPN supplemental injections. For modelling purposes, patients in the weekly phase of medication administration were defined as ‘initiating patients’. Those who had passed this phase – 'initiated patients’—were assumed to receive just a single administration per month [12, 13].

Methadone is supplied as an oral liquid in bottles. Individual doses are prepared by JHFMHN clinic nurses according to patients’ individual prescriptions administered daily.

### Study patient profile and cohort size

The number of study patients receiving depot BPN treatment in July 2019 was determined from the UNLOC-T trial records (*n* = 50), with just over one-third receiving the weekly preparation (*n* = 18; 36%); and the remainder (*n* = 32, 64%) treated with the monthly preparation. [17].

The costing study populations for the methadone and SL BPN-NLX comparator cohorts were estimated with JHFMHN data [28]. These data contained numbers of patients receiving each OAT medication in NSW government correctional facilities on the last day of the month (August 2018 to July 2019). The final patient numbers were calculated to be 239 and 40 for methadone and SL BPN-NLX, respectively.

### Identification, measurement and valuation of resource use

**Fig. 1:**
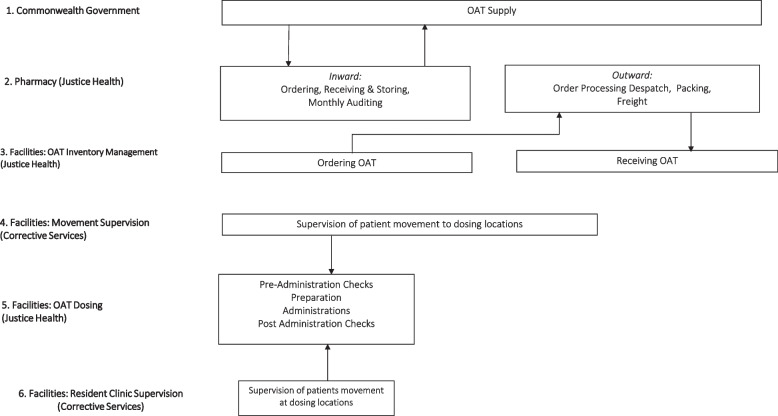
OAT Supply and administration pathway

For the purpose of identifying and measuring relevant cost parameters, lead authors (RL, BW) employed time-driven activity-based micro costing, directly observing medication administration at two correctional facilities. These observational data were supplemented with interviews conducted with key personnel across the trial centres (JHFMHN nursing, CSNSW custodial staff and JHFMHN pharmacy staff). All relevant individual costs were identified, valued, and aggregated for each OAT cohort and then compared [29]. Costs were collected for each of the cost centers of the OAT supply and administration process (Fig. 1).

Identified resource categories included: labour time and overheads associated with OAT administration and inventory management, consumables associated with medication administration (e.g. disposable gloves, dosing cups and kidney dishes), and labour time associated with OAT ordering, storing, dispatching, and transportation. Costs were measured and valued as *opportunity costs*, i.e. the costs of diverting these resources from alternative applications [30].

Unit costs and their sources are reported in Additional File 1. Wage rates were sourced from relevant wage awards, which are minimum wages legally publicly set by Australian industrial authorities [31–33]. All labour costs were scaled up by an additional 26% and 27.5% to account for ‘on costs’ (superannuation, leave loading and long service leave [34]) and overheads ((necessary operating expenses incurred but not directly measurable like electricity, water and building maintenance [18]), respectively. All nursing labour was costed for the rates of a Registered Nurse, Year 8 or above (RN8) as the regulation staff level for handling of OAT [31]. Custodial officer labour was costed at the wages rates of Correctional Officers 1st Class 2nd Year and above (CO 1, Year 2 and above) [32] a level commonly encountered during field observations. Pharmacy assistants were costs as Pharmacist Assistant (Grade 1, Year 8) and the senior pharmacist as Chief Pharmacist Group 1 & 3, Grade 5 (2^nd^ year, Corrections Health Service). The Pharmacy administrator was costed at the grade: Administrative and Clerical Officer Grade 6 [32]. Segregation administration was costed with the same preparation and checking times as the main group treatment events. Nurse time was further costed subjectively for 10 min of walking to and from each patient location. Materials costs (e.g. swabs, kidney dishes and gloves) were valued at market rates (Additional File 1). Costs of OAT were sourced from the Pharmaceutical Benefits Scheme (PBS) [35].

OAT costs were calculated according to quantities shipped to each facility, as documented by data provided by the JHFMHN Pharmacy for July 2019. These were costed at amounts advertised by the PBS [35]. These costs were averaged out across patients. Note that depot BPN is packaged in boxes each with a loaded syringe.

### Cost model structure

**Fig. 2:**
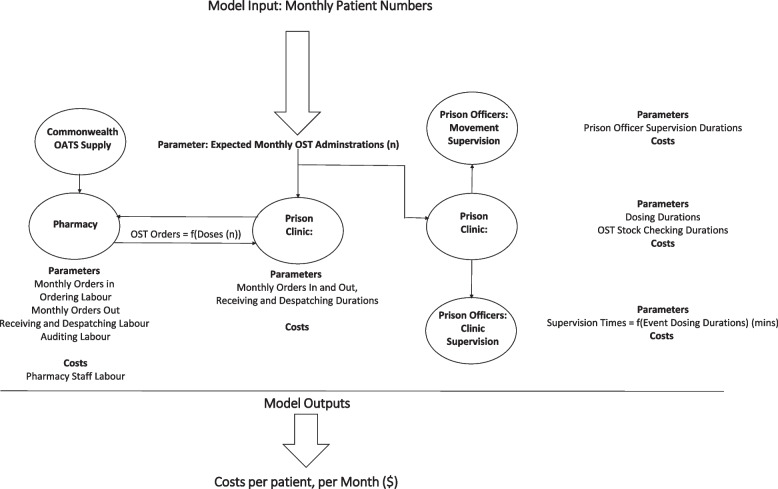
Cost model pathways

A cost model was created in a Microsoft Excel (2018) [36] structured around the cost centers of the OAT process (Fig. 1). Relationships between inputs and cost centers appear in Fig. 2 which represents the supply and administration of one OAT for one facility. The model replicates this flow for all facilities and OATs simultaneously.

Model outputs are produced for two scenarios: A base case which reflects the within trial dataset, and a second simulated scenario reflecting implementation of depot BPN over 12 months for the same aggregate patient population for all study facilities. The model assumed a steady incremental treatment transfer from methadone and SL BPN-NLX to depot BPN starting at 0% depot BPN. The percentage of OAT patients receiving depot BPN increased by 5% each month to reach 60% by month 12. The scenario reduces SL BPN-LX patients, whereby there are only two left by month 12. The authors note that some SL BPN-LX patients will likely continue to be in the NSW corrections system for valid clinical reasons.

The model also assumed that in their first month *initiating* patients transferring from methadone received two weekly depot BPN doses, before transfer to monthly dosing. SL BPN-NLX initiating patients were modelled as transferring immediately to monthly depot BPN administration, an expected regimen for an implementation. It was further assumed that 6.5 percent of all patients on weekly doses would receive one titration dose, as was found in the trial.

### Sensitivity analysis

Given uncertainty in some data and parameters, sensitivity analyses were conducted to test the robustness of the cost outputs given variation in input values. Table 2 presents the list of model parameters included in the analysis. Sensitivity analysis assigns the highest and lowest values reported during data collection for the given parameters. Percentage changes in monthly costs per patient for each medication are reported.

**Table 2:** Sensitivity analyses (each applied simultaneously to depot BPN, methadone, SBL BPN-NLX)

Scenario Group	Adjusted Variables	Variable Descriptions	Variation values^a^	
Activity Times^a^				
1	Inventory Ordering	Labour time creating OST orders for Pharmacy (mins)	Max	Min
2	Inventory Receiving	Labour time receiving OST orders from Pharmacy (mins)	Max	Min
3	Preparation and Clean Up	Labour time preparing administration event (mins)	Max	Min
4	Dosing Times^3^	Labour time dosing individual patients (mins)	Max	Min
5	Stock Checking Times	Labour time spot auditing OST stock levels (mins)	Max	Min
6	Pharmacy Dispensing	Labour time dispensing OAT for transport to facilities	+ 10%	-10%
Costs Rates^b^				
7	Overheads	Indirect costs (e.g. electricity, building maintenance), measured as a % of labour	+ 2.5% pts	-2.5% pts
8	Labour On-Costs	Labour costs outside the award wages e.g. superannuation, medical etc	+ 5% pts	-5% pts

^a^Max = maximum reported value applied to all sites; Min = minimum reported value applied to all sites

^b^Base Case: Overheads = 27.5% of labour costs; Labour On-Costs = 26.0% of labour costs

## Results

### Base case (within trial) cost outputs

Table 3 reports total costs and costs disaggregated by payer (JHFMHN CSNSW and Commonwealth). Monthly-per-patient medication administration and supply costs combined for NSW government services were calculated to as $151, $379 and $1,529 for depot BPN, methadone and SL BPN-NLX respectively. Commonwealth monthly medication costs per patient were similarly $434, $80 and $525 for depot BPN, methadone and SL BPN-NLX.

**Table 3:** Base Case Collected Data: Monthly Per-Patient Costs, July 2019

	Depot BPN	Methadone	SBL BPN
	($)	($)	($)
Justice Health & Forensic Mental Health			
Pharmacy	$24	$5	$32
Inventory Management	$26	$4	$16
Clinic OAT Administrations	$49	$208	$879
Total	$98	$216	$927
Corrective Services NSW			
Movement Supervision	$32	$72	$300
Clinic Supervision	$20	$91	$302
Total	$52	$162	$601
Total NSW Government ($)	$151	$379	$1,529
Patients (n)	50	239	40
Administrations (n)	84	7,409	1,240
Orders to Pharmacy (n)	21	24	24
Commonwealth Government			
OAT Supply Costs per patient	$434	$80	$525

### Simulated cost outputs

Monthly-per-patient medication administration and supply costs under the simulated scenario were calculated for depot BPN, methadone and SL BPN-NLX respectively. Table 4 reports total costs and costs disaggregated by payer (JHFMHN, CSNSW and Commonwealth).

**Table 4:** Depot BPN Implementation over 12 months

		**Depot BPN**								**Methadone**				
**Depot BPN Roll Out (% of all patients)**	**Implementation Month (#)**	**Patients (n)**	**% of All Patients**	**Patients on Weekly Doses**	**% Patients on Weekly Doses**	**Total Administrations (n)**	**Cost Per Patient ($)**			**Patients (n)**	**Total Administrations (n)**	**Cost Per Patient ($)**		
							**Justice Health**	**Corrective Services**	**Total**			**Justice Health**	**Corrective Services**	**Total**
**0%**	0	0	0%	0	0%	0	$0	$0	$0	282	8742	$214	$161	$374
**5%**	1	16	5%	13	81%	43	$134	$43	$178	269	8339	$214	$161	$375
**10%**	2	33	10%	13	39%	60	$96	$35	$131	256	7936	$217	$164	$381
**15%**	3	49	15%	13	27%	76	$86	$35	$120	243	7533	$222	$172	$394
**20%**	4	66	20%	13	20%	93	$81	$37	$117	230	7130	$219	$171	$391
**25%**	5	82	25%	13	16%	109	$74	$35	$109	217	6727	$225	$177	$402
**30%**	6	99	30%	13	13%	126	$70	$35	$105	204	6324	$232	$181	$412
**35%**	7	115	35%	13	11%	142	$69	$34	$103	191	5921	$235	$186	$421
**40%**	8	132	40%	13	10%	159	$67	$33	$101	178	5518	$242	$194	$437
**45%**	9	148	45%	13	9%	175	$65	$33	$98	165	5115	$251	$200	$451
**50%**	10	165	50%	13	8%	192	$63	$33	$96	152	4712	$256	$213	$469
**55%**	11	181	55%	13	7%	208	$62	$32	$94	139	4309	$269	$226	$494
**60%**	12	197	60%	12	6%	222	$60	$32	$92	127	3937	$282	$248	$530

		**SBL BPN-NLX**						OAT Costs		
**Depot BPN Roll Out (% of all patients)**	**Implementation Month (#)**	**Patients (n)**	**Total Administrations (n)**	**Cost Per Patient ($)**			**Total OAT Patients**	**Costs** **per patient**		
				**Justice Health**	**Corrective Services**	**Total**		**Depot**	**Methadone**	**SBL BPN-NLX**
**0%**	0	47	1457	$887	$602	$1,489	**329**	$0	$85	$525
**5%**	1	44	1364	$903	$585	$1,488	**329**	$531	$86	$526
**10%**	2	40	1240	$927	$568	$1,495	**329**	$442	$85	$526
**15%**	3	37	1147	$905	$619	$1,524	**329**	$422	$85	$525
**20%**	4	33	1023	$929	$600	$1,528	**329**	$409	$85	$525
**25%**	5	30	930	$958	$584	$1,542	**329**	$401	$85	$525
**30%**	6	26	806	$1,011	$576	$1,587	**329**	$394	$85	$525
**35%**	7	23	713	$1,054	$568	$1,622	**329**	$390	$85	$525
**40%**	8	19	589	$1,149	$576	$1,725	**329**	$388	$85	$525
**45%**	9	16	496	$1,156	$551	$1,707	**329**	$386	$85	$525
**50%**	10	12	372	$1,122	$536	$1,658	**329**	$385	$85	$525
**55%**	11	9	279	$1,284	$570	$1,855	**329**	$384	$85	$526
**60%**	12	5	155	$1,595	$567	$2,162	**329**	$381	$85	$525

Rounding errors apply

Table 4 shows results for the simulated implementation of depot BPN. In the pre-implementation month (month 0), total monthly-per-patient costs for methadone and SL BPN-NLX were calculated to be $374 and $1,489 respectively. As depot BPN is progressively introduced and assumed to comprise a growing percentage of all OAT patients, per patient costs for methadone and SL BPN-NLX steadily increase, as patient numbers decline against fixed costs. At six months, methadone and SL BPN-NLX costs respectively increase to $412 and $1,587 respectively. By twelve months, the expected monthly-per patient cost for methadone was calculated to be $530 and $2,162 for SL BPN-NLX, a figure driven by a small number of patients remaining on this treatment mode. The authors note that in practice, a small number of patients will continue to receive SL BPN-NLX, as clinically indicated.

Depot BPN monthly-per patient costs were highest when there was the greatest percentage of patients in the treatment initiation phase requiring two weekly and a monthly administration (i.e. three doses per month). Between month 1 and month 12, depot monthly cost per patent declined from $178 to $92.

### Sensitivity analysis results

**Fig. 3:**
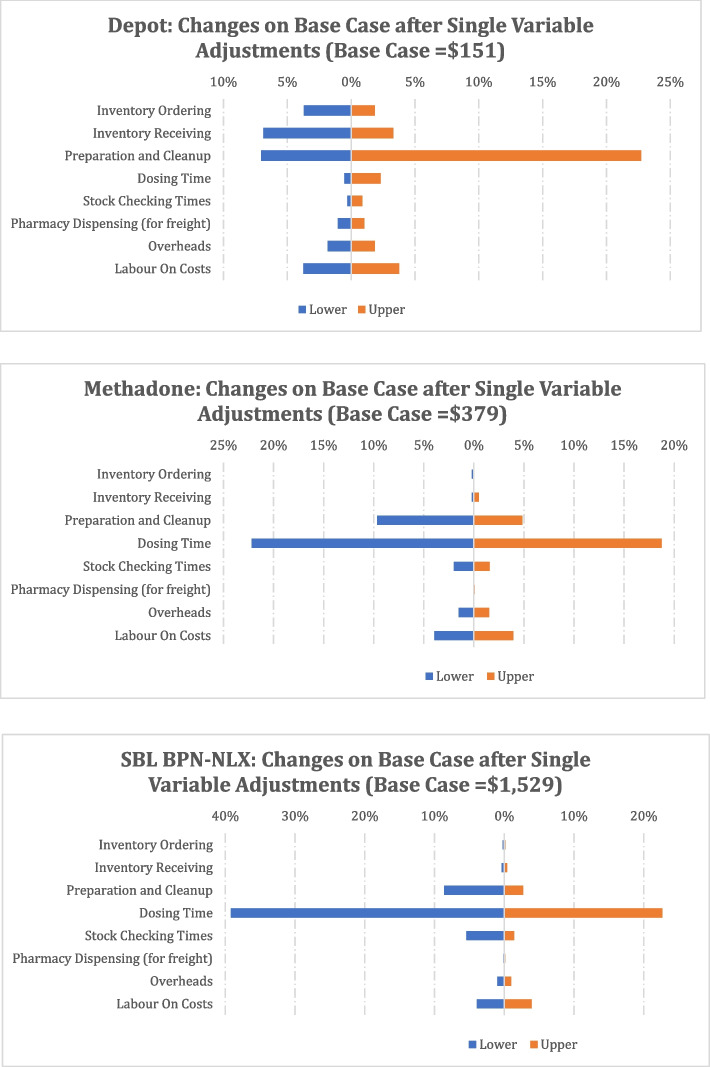
Sensitivity Analyses

Results for the sensitivity analysis are reported in Fig. 3 being based on data in Additional File 1. These results show that changes to assumptions and inputs impact the headline figures to a small extent only. The parameter to which headline results for methadone and SL BPN-NLX were most sensitive was found to be medication administration time. Depot BPN was sensitive to changes in preparation and clean up times, which occurred before and after each patient visit.

## Discussion

In this analysis the costs of methadone and SL BPN-NLX were higher than for depot BPN because each required one administration per day rather than one per month or week. SL BPN-NLX was associated with higher costs than methadone because: a) there were significantly fewer patients over which to spread fixed costs such as dosing preparation and inventory checking; and b) SL BPN-NLX patients commonly require several minutes to orally dissolve multiple sublingual films [37].

Simulation modelling showed steady decreases in monthly-per patient depot BPN costs as the percentage of fully initiated depot buprenorphine patients increased. Sensitivity analysis showed that total costs were most sensitive to treatment administration time.

Concern regarding diversion in custodial populations, particularly of SL BPN-NLX, is emphasized in NSW Ministry of Health guidelines [38]. Diversion may lead to an increase in opioid use and dependence; and patients found diverting may have their treatment discontinued [6]. It is also associated with prison violence, while those receiving diverted opioids will inject—sometimes oral or sublingual intake—without supervision [38, 39]. Potential societal costs can be considered in terms of increasing numbers of patients exiting the corrections system with continued addiction problems and increased chances of recidivism. Families, crime victims and law enforcement institutions would potentially sustain emotional and financial costs.

Given depot BPN is administered by injection, the risk of diversion is expected to be lower [38, 40]. This is supported by the trial results which showed no increased risk of diversion [17]. Whether widespread implementation of depot BPN critically reduces OAT diversion in NSW corrections facilities is a topic for future research [17]. However, with the findings of this study, future implementation decisions will be informed of its cost advantages over the standard alternatives.

### Limitations

As a cost modelling study this analysis has several limitations. First, the patient populations forming the basis of the calculations for methadone and SL-BPN-NLX were based on estimates (including a monthly census of the number of patients in treatment on the last day of each month over the previous 12 months and staff estimations), rather than the specific number of patients that received each treatment type at each facility in July 2019. This information was not readily available. Second the study could only feasibly extract pharmacy data for one month. A larger observation period would have allowed more accurate matching of patient flows between patient numbers and pharmacy supplies. Further, the study used a small sample of participating centres (*n* = 7). The study also excluded OAT associated costs such as management of adverse events and treatments for substance use related morbidities like hepatitis and HIV.

The model excludes some OAT related costs. These are costs, of OAT associated with OAT diversion or related assaults, medical officer time in assessing patients to commence treatment, regular review during treatment or reviews about dose titrations [22]. Further, the researchers found no proxy for availability of clinical space – a known limited resource. Central clinical and administration labour needed to coordinate the program and undertake administrative and regulatory reporting to Ministry of Health were also excluded [22]. However, information provided from JHFMHN staff suggested that the resources expended in these areas would the same per patient for all comparator treatments. Other cost omissions were adverse events, downstream health care costs, hazardous waste disposal costs, and disposables such as band aids, that may be used in other settings for patients receiving depot BPN.

## Conclusion

This study modelled the comparative costs of depot BPN, methadone and SL BPN-NLX, based on data collected from seven NSW correctional centres. Both within trial analysis and simulated analysis, assessing scale up of depo BPN treatment access, found consistent cost advantages for depot BPN. This study provides comparisons of resource inputs and volumes used in the administration of each medication. Such findings inform decisions related to the use of depot BPN; and strategies for cost improvement across all three medications. The results can also be used by other correctional systems, with adjustments for their own conditions. Future cost-effectiveness modelling can use the results as a source of data.

Future research could usefully expand on the scope of this analysis by addressing the data gaps described above and also assessing the extent to which widespread implementation of depot BPN improves access to OAT treatment as well as critically reducing diversion.

## Supplementary Information

**Additional file 1. **

**Additional file 2. **CHEERS checklist.

## Data Availability

Part of the data was sourced from the UNLOC-T (registration and ethics approvals details above). The data that supports the findings of this study are available from the authors, but restrictions apply to the availability of these data, which were used under license for the current study, and so are not publicly available. Data are however available from the authors upon reasonable request and with permission of the corresponding author.

## Abbreviations

OAT: Opioid agonist treatment
SL BPN-NLX: Sublingual buprenorphine-naloxone
UNLOC-T: ‘Understanding NSW Long-acting Opioids in Custody-Treatment’
depot BPN: Depot buprenorphine
JHFMHN: Justice Health and Forensic Mental Health Network
AH&MRC: Aboriginal Health and Medical Research Council
CSNSW: Corrective Services NSW
ANZCTR: Australia New Zealand Clinical Trials Registry
PBS: Pharmaceutical Benefits Scheme
